# Dataset on coherent control of fields and induced currents in nonlinear multiphoton processes in a nanosphere

**DOI:** 10.1038/sdata.2015.64

**Published:** 2015-11-24

**Authors:** Duncan McArthur, Ben Hourahine, Francesco Papoff

**Affiliations:** 1 Department of Physics, SUPA, University of Strathclyde, 107 Rottenrow, Glasgow G4 0NG, UK

**Keywords:** Nanophotonics and plasmonics, Nonlinear optics

## Abstract

We model a scheme for the coherent control of light waves and currents in metallic nanospheres which applies independently of the nonlinear multiphoton processes at the origin of waves and currents. Using exact mathematical formulae, we calculate numerically with a custom fortran code the effect of an external control field which enable us to change the radiation pattern and suppress radiative losses or to reduce absorption, enabling the particle to behave as a perfect scatterer or as a perfect absorber. Data are provided in tabular, comma delimited value format and illustrate narrow features in the response of the particles that result in high sensitivity to small variations in the local environment, including subwavelength spatial shifts.

## Background & Summary

Recently several groups have been able to enhance light-matter interaction processes by controlling the near and far field optical response of nanostructures. Control methods include nonlinear^1^ and linear control based on pulse shaping^2,3^, combination of adaptive feedbacks and learning algorithms^4^, as well as optimization of coupling through coherent absorption^5^, time reversal^6^ and phase and polarization control^7^. Spatiotemporal control of surface plasmons in nanosystems has been described using ultrashort pulses^8–11^. Interference between fields was proposed in quantum optics as a way to suppress losses in beam splitters^12^ and has been recently applied to show control of light with light in metamaterials^13,14^, and in graphene films^15^. Coherent control of second-harmonic generation using a second pump beam has been recently demonstrated numerically in particles with cylindrical symmetry^16^. For spheres, it was shown in ref. 17 that the directionality of the emission obtained combining two pump beams results from selection rules that depend on the order of specific process and on the size of the particles.

In a recent paper^18^, we model a scheme for the coherent control of scattering and absorption patterns in a nanosphere in a uniform background which applies independently of the multiphoton processes at the origin of scattering and absorption, as long as the pump beam is not depleted. We use a control beam coherent with the radiation produced by the nonlinear process: a simple way to realize this is by driving two nonlinear processes of the same order with the same pump, using the output of one of them to control the other. Using the Huygens-Fresnel principle, formally proved in the Stratton-Chu theorem^19^, we can understand this scheme in terms of the formation of equivalent surface currents, which are combination of physical surface currents proportional to surface polarizations and tangent field components. These are due to the control field, incident to the surface of the sphere from the outside, and to the field generated by the nonlinear volume polarization, when this is present, which is incident to the surface from the inside. Forming equivalent surface currents that can radiate only outside or inside the particle we induce the particle to behave as a perfect scatterer or a perfect absorbed on the controlled modes. These equivalent surface currents depend linearly on the control field, so this is a linear control scheme.

The control is extremely sensitive to phase variations and produces a reduction of the absorption and variations in the scattered energy of several orders of magnitude. These features can be applied to detection of changes in the position of the particle far smaller than the particle itself, suppression of radiative losses, sensing of variations in the electric permittivity, *ϵ*, and magnetic permeability, *μ*, and optical switching.

For applications in which substrates are used, the theory as it stands can be applied only when the index between the substrate and the medium that contains the spheres is matched, and the thickness of the substrate is such that reflections from the lower face of the substrate and guided modes in the substrate can be neglected. When these conditions are not met, substrates remove the reflection symmetry and perturb the modes of the particle reducing the degeneracy among them^20,21^. From the point of view of applications, this is actually a beneficial effect, as degeneracy makes selecting the correct angles of incidence and observation more difficult and requires the use of a larger number of control beams. On the other hand, the theory will have to be performed using the modes of the particles in presence of the substrate and not the Mie’s modes used in this paper.

When pump and control beams with broad spatial profiles are used, the relative phase differences are (almost) spatially periodic over the cross section of the pump, so that the optimal control conditions will be formed on an array of spatial points. On points in this array that are separated by at least a wavelength, the theory used here can be extended also to the control of arrays of spheres in which the interaction among different spheres is negligible. Control of arrays of interacting particles is also possible, but in that case the theory will have to be adapted by considering the modes of the array.

With appropriate control beams and pump, one can control the directionality of nonlinearly generated electromagnetic waves not only in a single sphere, but also in a regular array of spheres, for which both the radiation patterns and the spatial positions could be determined. This can be very useful for applications such as optical antennae and for surface enhanced spectroscopy, providing a reference of regularly spaced optical nano beacons for the localization of molecules.

The data stored in the repository, access details are provided in Data Citation 1, allows one to verify and test the results shown in the figures published in the Scientific Report paper^18^ and in this paper.

## Methods

The theory behind this work is explained in detail in a Scientific Reports paper by the same authors^18^ and relies on the ability of determining the effect of both surface and volume nonlinearities by considering the boundary conditions at the surface of the sphere. Here we give the equations necessary to reproduce the results published in that paper. Surface^22,23^ and volume nonlinearities appear in the boundary conditions at frequency *ω* as

(1)εinE⊥i−εexE⊥s=−εinE⊥B+εexE⊥c−∇∥⋅PS,

(2)E∥i−E∥s=−E∥B+E∥c−(εex)−1∇∥P⊥S,

(3)H∥i−H∥s=−H∥B+H∥c+iω(nˆ×PS),

where *E* and *H* are the electric and magnetic fields, respectively, *i*, *s*, *c* stand for internal, scattered and external control fields, *ex*, *in* for external and internal, and $E_{⊥}=\hat{n}(\hat{n}\cdot E)$, $E_{∥}=-\hat{n}\times (\hat{n}\times E)$ and analogously for the other fields. *E*^*i*^ and *E*^*s*^ are the combination of particles modes (solutions of the homogeneous equations without nonlinear polarizations) that fulfill the boundary conditions. The modes’ amplitudes depend upon the left-hand sides of equations (1,2,3) which, for any *E*^*B*^, *H*^*B*^ and *P*^*S*^, enable us to find the form of *E*^*c*^, *H*^*c*^ necessary to control the interaction of light with the particle through the amplitudes of the internal and scattering modes, regardless of the nature of the underlining nonlinear processes.

For sake of simplicity, we concentrate here the control of two modes and outline later how the theory generalizes to an arbitrary number of modes. As a consequence of the rotational invariance, the only modes that are spatially correlated at the surface of a sphere are internal and scattering electric or magnetic multipoles with the same value of *l* (total angular momentum) and *m* (angular momentum along the direction of propagation of the pump). Electric (magnetic) multipoles have magnetic (electric) fields with null radial component^24^. We recall that there are another two types of multipolar waves for the external medium that are relevant to this work: incoming, which propagate inward and have a divergence at the center, and regular, which are used to expand waves with amplitudes bounded everywhere, as the plane waves. All types of electric or magnetic multipoles with the same indexes *l* and *m* have the same angular dependence in spherical coordinates^24^, but different radial dependence. In our notation

(4)fc=(εexE⊥c,E∥c,H∥c),

(5)fNL=−(εinE⊥B+∇∥⋅PS,E∥B+(εex)−1∇∥P⊥S,H∥B−iωnˆ×PS),

are the surface vector functions of the control field (*f*^*c*^) and of the nonlinear (*NL*) sources that appear in the boundary conditions, equations (1,2,3), for a pump of amplitude *a*^*p*^=1 in arbitrary units. The real amplitude and phase of *f*^*c*^ are encoded in the complex amplitude *a*^*c*^. For any pair of internal and scattering modes, *i*_*lm*_, *s*_*lm*_, for which we adopt the same notation as for *f*^*c*^, the amplitudes $a_{lm}^{i},a_{lm}^{s}$ are given by

(6)[almi−alms]=[i′lm⋅fci′lm⋅fNLs′lm⋅fcs′lm⋅fNL][acaNL]

where the scalar product indicates the sum of the overlap integrals (i.e., the spatial correlations) of all the components with *a*^*NL*^=(*a*^*p*^)^*N*^ the amplitude of *f*^*NL*^ and *N* the order of the nonlinear process. Note that *s*_*lm*_, *i*_*lm*_, are either transverse electric or transverse magnetic, but for ease of notation we do not specify which type they are. The biorthogonal mode^25^ ${s\prime }_{lm}\;({i\prime }_{lm})$ is orthogonal to all modes other than *s*_*lm*_ (*i*_*lm*_). For spheres the biorthogonal modes can be found analytically and depend on all internal and scattering modes with the same *l* and *m*, correlated at the surface of the sphere, according to the formula

(7)u′j=uiGij−1,

where *u*_1_=*s*_*lm*_, *u*_2_=*i*_*lm*_, *G*^−1^ is the inverse of the (Gram) matrix with elements *u*_*ij*_=(*u*_*i*_·*u*_*j*_) and we sum over repeated indexes. When longitudinal modes are present^26^, we can include them simply by defining *u*_3_ as the longitudinal mode spatially correlated to *s*_*lm*_ and *i*_*lm*_.

Generalizing equation (6) to include any number of modes and external incident waves is straightforward, as the amplitude of each mode requires only the scalar product of its biorthogonal mode with the sum of all the fields incident on the surface and the surface polarization. For any set of incident electromagnetic waves, $\left\{f_{j}^{ex}\right\}$, the first column of the matrix in equation (6) is replaced by two matrices: the matrix *S* with elements $S_{ij}=-{s\prime }_{i}\cdot f_{j}^{ex}$ and the matrix *I* for the internal modes with elements $I_{ij}={i\prime }_{i}\cdot f_{j}^{ex}$, where *i*=(*l*, *m*). When *f*^*NL*^=0, the amplitudes of the modes are given by the product of these two matrices with the amplitudes of the incident waves. When *f*^*NL*^≠0, the amplitudes of the modes are given by the product of the augmented matrices $\tilde{S}$ and $\tilde{I}$, with $\tilde{S}\;(\tilde{I})$ obtained by adding to *S* (*I*) the column $-{s\prime }_{i}\cdot f^{NL}\;({i\prime }_{i}\cdot f^{NL})$, with a column vector containing the amplitudes of the incident waves and of *f*^*NL*^. Control of the amplitudes of *N* modes can be achieved with *N*−1 control beams when *f*^*NL*^≠0 and the matrix ${\left[\tilde{I},\tilde{S}\right]}^{T}$ is invertible.

### Code availability

We used the Fortran90 code Sphere.f90, version 347, which calculates the formulae given above evaluating spherical Bessel and Hankel functions using subroutines supplied with the book A. Doicu *et al.*, Light Scattering by Systems of Particles, Springer (2006)^27^. We are happy to pass on the part of this code that we wrote to other researchers who can then use it if they have the required subroutines. We cannot provide these subroutines due to copyright restrictions.

## Data Records

Numerical data have been generated with a custom Fortran90 code and are available on PURE, see Data Citation 1, the repository of the University of Strathclyde. For the control of a gold sphere, we have used the previous analytical equations and the Lorentz-Drude model for the for the dielectric function of gold. Data are given in tabular, comma delimited value format, with columns named according to the quantity plotted in the corresponding figures of the Scientific Report paper^18^ and in this paper.

LD.csv contains data for the dielectric function of gold calculated with a Lorentz-Drude model^28^.

We control the internal and scattering modes *i*_10_ and *s*_10_ of the electric dipole to generate data in Fig2a.cvs and Fig2b.cvs. In Fig2a.cvs the amplitude of the control beam is chosen so that the amplitude of *s*_10_, $a_{10}^{s}$, can vanish at the appropriate phase; data columns are the intensity of the field scattered in a direction orthogonal to both pump and control: other multipoles do not emit in this direction so the intensity has the same dependence of the amplitude $a_{10}^{s}$ and shows an extremely sharp variation.

The ratio of the amplitudes $a_{10}^{s}$ and $a_{10}^{i}$ shows that we find the condition for a perfect scatterer in Fig2a.cvs and for a perfect absorber in Fig2b.cvs, while the amplitudes of the other modes are not affected by the control beam. By removing the dominant internal mode, we can minimize the total absorption, which is very useful to reduce heating and, as a consequence, increase stability in experiments.

Data in Fig3.cvs shows the radiation patterns with and without control in the equatorial plane *θ*=90° of the sphere.

In Fig4a.cvs, we give data for the intensity of the field scattered in a direction at *π*/2 with respect to the control beam and at *π*/4 with respect to the pump. In Fig4b.cvs we give the amplitudes on the modes excited, showing that the control beam affects only the modes *l*=2, *m*=±2. Even in this case we can observe a subwavelength variation of the intensity.

In Fig5a.cvs we give the intensity scattered in the same direction as for data in In Fig4a.cvs, but using an incoming multipolar wave with *l*=2, *m*=2 as control beam. In this case the variation of the intensity is smaller than in Fig4a.cvs because the multipolar control wave affects only the *l*=2, *m*=2 mode, as can be seen by plotting data in Fig5b.cvs. This shows that using incoming multipolar waves (which are extremely hard to realize experimentally) is not necessarily more effective than using plane waves. Finally, plotting data in Fig6a.cvs and Fig6b.cvs shows how the sensitivity to phase variation can be applied to monitor small variations in the dielectric permittivity of the host medium; similar results could be achieved with variations of the magnetic permeability. With the intensity and phase of the pump and control beams optimised to suppress the *s*_10_ mode for a particular environment, *ϵ*^*ex*^, (corresponding to Δ*ϵ*^*ex*^=0 in Fig. 6) we observe a strong sensitivity to small changes in *ϵ*^*ex*^ in the scattered intensity. As the modes of the system depend upon the local environment, the relative phase and amplitude of the control beam required to maintain suppression of the modes change with it. When we vary the optimised amplitude of the control field by ±20% we observe in Fig6a.cvs that the curve of the scattered intensity drifts, so that the minima no longer occurs at Δ*ϵ*^*ex*^=0, and the sensitivity decreases slightly. In Fig6b.cvs we observe that the sharpness of the feature in the scattering intensity reduces significantly when the relative phase of the control beam, $\Phi^{c}$, is changed from the optimised value, but the position of the minima in this case does not drift.

Finally, we give the data used to validate the numerical code and plotted in the figures in this data descriptor.

spheres-dielec.csv contains values for the complex dielectric function of gold^29^, found using a fit to data in P.B. Johnson and R.W. Christy^30^, used to generate the following files.

spheres.csv contains the data for the extinction efficiencies against wavelength for gold spheres of radii *r*=10, 25, 50 and 400 nm, in a host medium of water (*n*=1.3), calculated using the code bhmie.f, see below, with the fitted dielectric function for gold.

spheres-test.csv contains the data for the extinction efficiencies against wavelength for gold spheres of radii *r*=10, 25, 50 and 400 nm, in a host medium of water (refractive index *n*=1.3), calculated using our sphere code with the fitted dielectric function for gold.

## Technical Validation

**Figure 1:**
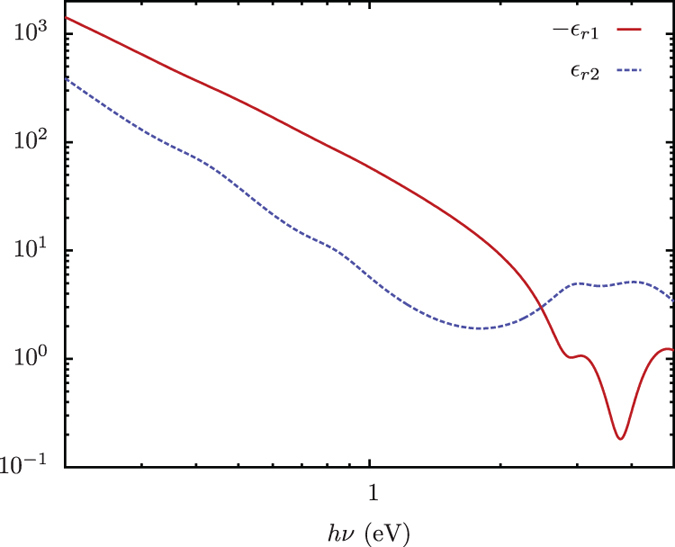
The components of the complex dielectric function for gold calculated using a Lorentz-Drude oscillator model^28^. The real $\left(\varepsilon_{r1}\right)$ and imaginary $\left(\varepsilon_{r2}\right)$ parts of the optical dielectric function plotted against photon energy (*hν*) in electron volts, where *ν* is the frequency.

The datasets referenced in this descriptor were validated via comparison with results from literature. All calculations were performed for gold particles using a Lorentz-Drude oscillator model for the complex dielectric function^28^,

(8)εr(ω)=εr1(ω)−iεr2(ω)

(9)=1−f0ωp2ω(ω−iΓ0)+∑j=1kfjωp2(ωj2−ω2)+iωΓj,

where *ω* is the frequency, $\omega_{p}$ is the plasma frequency, *k* is the number of oscillators and *f*_*j*_ is the oscillator strength, $\omega_{j}$ is the oscillator frequency and 1/Γ_*j*_ is the oscillator lifetime. The terms with *j*=0 are associated to the intraband transistions. This model, including the relevant values for the oscillator strength, frequency and damping, were taken from^28^ and written as a custom Fortran90 code. The output of this code was validated by reproducing the values of the complex dielectric function, plotted against oscillation energy, in ref. 28. The results are presented in Fig. 1.

**Figure 2:**
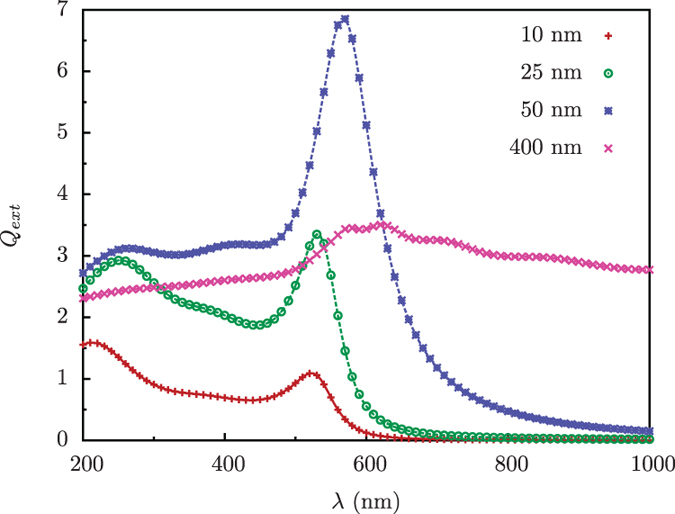
Extinction efficiency spectra for spheres of different radius. The calculations were performed for a linear system with local response using a dielectric function fitted from data in the literature. For spheres of radius *r*=10, 25, 50 and 40 nm in a host medium of water (*n*=1.3), the extinction efficiencies *Q*_*ext*_ via plane wave excitation calculated using the code written by Bohren and Huffman^31^ (points) were compared with our own results (lines).

The linear part of the numerical code used to calculate the fields for a system with local response was validated against the Mie code written by C.F. Bohren and D.R. Huffman (bhmie.f) in ‘Absorption and Scattering of Light by Small Particles’, New York, Wiley, (1983)^31^, which is widely available online. We calculated the extinction efficiencies defined as,

(10)Qext=σextπ(rλ)2,

where *ω* is the wavelength of the incident field, *r* is the particle radius and *σ*_*ext*_ is the extinction cross-section. For a direct comparison of the results, we normalize the values we calculated by a factor of $4\pi^{3}$. The (normalized) values calculated by both codes are plotted in Fig. 1; Fig. 2.

## Usage Notes

We have used Gnuplot for plotting the data, but any software package able to read data in csv (comma separated value) format should produce the same results.

## Additional Information

**How to cite this article:** McArthur, D. *et al.* Dataset on coherent control of fields and induced currents in nonlinear multiphoton processes in a nanosphere. *Sci. Data* 2:150064 doi: 10.1038/sdata.2015.64 (2015).

## Supplementary Material
